# Entre el olvido y el recuerdo de la pandemia de gripe de 1918-1919 en tiempos de la covid-19

**DOI:** 10.1590/S0104-59702023000100057

**Published:** 2023-10-23

**Authors:** María-Isabel Porras

**Affiliations:** i Facultad de Medicina de Ciudad Real Universidad de Castilla-La Mancha Ciudad Real Castilla-La Mancha España mariaisabel.porras@uclm.es Catedrática de Historia de la Ciencia, Facultad de Medicina de Ciudad Real/Universidad de Castilla-La Mancha. Ciudad Real – Castilla-La Mancha – España mariaisabel.porras@uclm.es

La denominada gripe española constituyó una grave experiencia para la población en su conjunto y un reto para la medicina. Para los profesionales sanitarios tuvo un impacto que perduró más allá de los años pandémicos. Fue determinante para promover el establecimiento del Programa de Lucha contra la Gripe de la Organización Mundial de la Salud, apoyado en la creación del World Influenza Centre (Londres) y una red de laboratorios regionales y nacionales en torno a este centro. La red, modelo para otras patologías (Porras Gallo, 2020), sigue siendo clave para monitorizar los virus influenza circulantes, detectar cambios relevantes y decidir la composición de las vacunas antigripales anuales. Sin embargo, el interés de los historiadores por esta pandemia es relativamente reciente. Quedó encubierta por la atracción que suscitó la Primera Guerra Mundial, adquirió protagonismo durante algunas crisis sanitarias posteriores y, sobre todo, con la emergencia del sida y el cuestionamiento del fin de la patología infecciosa.

Los trabajos publicados desde finales de 1970 dieron paso en los 1990 a un aumento progresivo de estudios, desde variadas perspectivas, sobre el desarrollo de esta pandemia, las respuestas articuladas y su impacto posterior en puntos geográficos distintos. Cuando cumplió 80 años, impulsó la celebración de jornadas de reflexión histórica sobre este suceso, que se repitieron 10 años después y en su centenario, dando lugar a varias obras colectivas (Phillips, Killingray, 2003; Sobral et al., 2009; Porras-Gallo, Davis, 2014). Estas reuniones y los estudios contenidos en dichas obras han permitido ampliar las fuentes utilizadas y las miradas dirigidas, consolidar nuevos formatos de investigación, visualizar la evolución historiográfica y conceptual que se producía en el estudio de la pandemia de gripe de 1918-1919, y evidenciar la complejidad de estos sucesos y su condición de crisis social global.

Este libro (Beiner, 2021) contribuye a enriquecer esta literatura con un conjunto de ensayos que tratan de clarificar la disonancia entre la gran magnitud de la pandemia de gripe de 1918-1919 y el aparente eclipse de su memoria, tradicionalmente atribuido a su proximidad a la Primera Guerra Mundial (p.2). Se apoyan para ello en la exploración de un conjunto de historias personales, comunitarias, médicas y culturales procedentes de diferentes marcos nacionales y transnacionales con la intención de desvelar la memoria conservada sobre la grave crisis sanitaria, el olvido, los silencios y la reemergencia de los recuerdos latentes. Esta publicación deriva del proyecto de investigación “Forgetting and remembering the Great Flu: Laying the foundations for a global-transnational history of cultural amnesia and rediscovery” (“Olvidar y recordar la Gran Gripe: sentando las bases para una historia global-transnacional de la amnesia cultural y el redescubrimiento”), financiado por la Israel Science Foundation y dirigido por el editor del libro. También emana de dos reuniones internacionales, organizadas bajo los lemas “Cultural histories of the great flu pandemic of 1918-1919: Representations and memories” (“Historias culturales de la gran pandemia de gripe de 1918-1919: representaciones y memorias”) y de “Forgetting, remembering and rediscovering the Great Flu” (“Olvidar, recordar y redescubrir la Gran Gripe”), que tuvieron lugar, respectivamente, en febrero y diciembre de 2019 y reunieron a investigadores internacionales especialistas en la pandemia de gripe de 1918-1919. La experiencia de la pandemia de covid-19 ha enriquecido sustancialmente los resultados de este libro.

El editor y la gran mayoría de los autores de los capítulos son historiadores e historiadoras especializados en distintos periodos históricos y acercamientos (historia social, historia cultural, historia de la medicina etc.). Dos periodistas científicos y una conservadora de museo completan el equipo. El editor del libro y los otros autores comparten una formación académica obtenida en el mundo anglosajón, donde la mayoría desarrolla o ha desarrollado su actividad profesional. Únicamente tres de los investigadores poseen una larga trayectoria en el estudio de la gripe de 1918-1919, iniciada en las décadas de 1970 o 1980. Los 19 restantes se interesaron por la pandemia con motivo del desarrollo del síndrome respiratorio agudo grave (2003), la pandemia de gripe de 2009-2010 y, sobre todo, del centenario de esta crisis sanitaria.

Los casos particulares reunidos en esta obra muestran distintos procesos de construcción social de la memoria y del olvido sobre la gripe de 1918-1919 efectuados en los años pandémicos e inmediatamente posteriores en los entornos geográficos y culturales seleccionados. También revelan algunas iniciativas y/o circunstancias que han contribuido a efectuar nuevas construcciones sociales y a recuperar la memoria sobre la crisis sanitaria. Son varios los trabajos que dan cuenta del papel estimulante desempeñado por el centenario de su surgimiento y la experiencia de la covid-19 para despertar el interés particular y construir una memoria colectiva sobre la aparentemente olvidada gripe española. Es interesante señalar cómo varios ensayos ponen de relieve la permanencia de la pandemia en los recuerdos individuales y familiares que se transmitieron oralmente a los descendientes y quedaron protegidos del olvido oficial o del olvido cultural. El estudio del arte y la literatura modernista de algunos países europeos ha revelado cómo hay muchas obras canónicas sobre la contienda mundial, mientras la gripe estuvo ausente del canon literario y artístico, pero permaneció en el recuerdo individual. Son varios los trabajos que corroboran el papel de la Primera Guerra Mundial en la construcción del olvido colectivo de la gripe de 1918-1919 en algunos países, siendo común la ocultación de las muertes porque no eran heroicas como las de la contienda bélica. Otros trabajos desvelan el peso que tuvieron las circunstancias locales en ese proceso. Uno de estos casos, expuesto por E. Thomas Ewing en el capítulo 14, fue la Unión Soviética, donde la concurrencia de otras enfermedades, hambre, la guerra civil y las acciones para mejorar la atención sanitaria y la salud pública oscurecieron la memoria individual de la pandemia de gripe. Un capítulo particularmente interesante es el de Utz Thimm sobre el recuerdo de la pandemia en los Países Bajos por cuanto muestra cómo se construyó la memoria pública oficial sobre la crisis sanitaria en Bélgica y Holanda de modo distinto, estando más presente en este último país que en Bélgica, donde se prestó más atención a la Primera Guerra Mundial. Sin embargo, los belgas guardaron la experiencia de la gripe de 1918 en la memoria individual, como mostró la pandemia de gripe de 2009. Además, en los territorios coloniales de ambos países la experiencia pandémica fue mayor que en las metrópolis, y, ante la falta de interés mostrada por esas realidades, se guardó en la memoria a través del folclore popular de las colonias. Una aportación importante de este libro es destacar el dinamismo del proceso de construcción social de la memoria, del olvido y del despertar sobre la pandemia de gripe de 1918-1919 a lo largo de una centuria.

Creemos que el libro presentado es de gran interés para historiadores especialistas en la pandemia analizada, pero también para investigadores sobre la historia de otras enfermedades. Sin duda, esta obra abre nuevas vías de análisis de los sucesos epidémicos, pero también creemos que sería interesante ampliar en un futuro el fructífero enfoque adoptado con otros estudios de casos centrados también en la pandemia de gripe de 1918-1919 en zonas geográficas menos representadas en este libro, tanto de Europa como de Latinoamérica, África y Asia.
