# Supplementary material for: Entre el olvido y el recuerdo de la pandemia de gripe de 1918-1919 en tiempos de la covid-19
Source: Hist Cienc Saude Manguinhos. 2023 Oct 23;30:e2023057. [Article in Spanish] doi: 10.1590/S0104-59702023000100057 (PMC10593374; doi:10.1590/S0104-59702023000100057)
Supplement: Translation

# Translation: Between forgetting and remembering: the flu pandemic of 1918-1919 in the time of covid-19

**DOI:** 10.1590/S0104-59702023000100057en

**Authors:** María-Isabel Porras

**Affiliations:** i España Catedrática de Historia de la Ciencia, Facultad de Medicina de Ciudad Real/Universidad de Castilla-La Mancha. Ciudad Real – Castilla-La Mancha – España mariaisabel.porras@uclm.es mariaisabel.porras@uclm.es

**Figure:** BEINER, Guy (ed.). *Pandemic re-awakenings: the forgotten and unforgotten “Spanish” flu of 1918-1919*. New York: Oxford University Press, 2021. 391p.

The so-called Spanish flu was a traumatic experience for the population as a whole, and a challenge for medicine and health professionals, with an impact which lasted beyond the years of the pandemic. It played a decisive role in promoting the establishment of the World Health Organization Influenza Control Programme, supported by the creation of the World Influenza Centre (London) and a network of regional and national laboratories to deal with it. This network, a model for other pathologies (Porras Gallo, 2020), is still the key to monitoring the circulation of influenza viruses, detecting relevant changes, and deciding the composition of annual influenza vaccines. However, the interest of historians in this pandemic is relatively recent. It was overshadowed by the attention given to the First World War, acquired prominence during certain subsequent health crises and, above all, with the emergence of aids and the questioning of the end of infectious diseases.

Works published since the late 1970s gave way in the 1990s to an increasing number of studies from a variety of viewpoints, on the development of this pandemic, responses to it, and its subsequent impact in different geographical locations. Its 80th anniversary prompted seminars reflecting on the historical aspects of the event, which were repeated 10 years later and again on the centenary, giving rise to several joint books (Phillips, Killingray, 2003; Sobral et al., 2009; Porras-Gallo, Davis, 2014). These meetings and the contributions contained in the books have made it possible to broaden the sources used and the frames of reference, to consolidate new research formats, to see the historiographical and conceptual evolution of the study of the 1918-1919 flu pandemic, and to demonstrate the complexity of the events and their status as a global social crisis.

This book (Beiner, 2021) helps to enrich the existing literature with a compilation of essays that seek to clarify the discrepancy between the magnitude of the 1918-1919 influenza epidemic and the apparent eclipse of its memory, traditionally ascribed to its proximity to the First World War (p.2). They do so by exploring a collection of personal, community, medical and cultural stories from different national and transnational backgrounds, with the aim of revealing the surviving memories of the acute health crisis, the oblivion, the silence, and the re-emergence of latent memories. This publication has its origins in the research project entitled “Forgetting and remembering the Great Flu: Laying the foundations for a global-transnational history of cultural amnesia and rediscovery,” financed by the Israel Science Foundation and directed by the editor of the book, and in two international meetings organised under the titles “Cultural histories of the Great Flu pandemic of 1918-1919: Representations and memories” and “Forgetting, remembering and rediscovering the Great Flu,” which took place in February and December of 2019 respectively, bringing together international researchers specialising in the influenza pandemic of 1918-1919. The experience of the covid-19 pandemic has substantially enriched the results of this book.

The editor and the great majority of the authors of the chapters are historians specialising in different historical periods and approaches (social history, cultural history, history of medicine etc.). Two science journalists and a museum curator complete the team. The editor of the book and the other authors share an academic background obtained in the Anglo-Saxon world, where most of them exercise, or have exercised, their professional activity. Only three of the researchers have an extensive background in the study of the 1918-1919 influenza, beginning in the 1970s or 1980s. The other 19 became interested in the pandemic as a result of the development of the severe acute respiratory syndrome (2003), the flu pandemic of 2009-2010 and, above all, the centenary of that health crisis.

The individual cases included in this work show the different processes of social construction of memory and oblivion about the 1918-1919 flu that took place in selected geographical and cultural environments in the years of the pandemic and immediately afterwards. They also reveal initiatives and/or circumstances that have contributed to creating new social constructs and to recovering the memory of the health crisis. Several of the works explain the stimulating role played by the centenary of its emergence and the experience of covid-19 in arousing individual interest and in building a collective memory about the apparently forgotten Spanish flu. It is interesting to note how several essays highlight the way that the pandemic remained in individual and family memories, which were transmitted orally to descendants and were protected from official oblivion or cultural oblivion. The study of the modernist art and literature of some European countries has shown that there are many canonical works on the world war, while the flu was absent from the literary and artistic canon but remained in the individual memory. Several of the articles corroborate the role of the First World War in the construction of the collective oblivion of the flu of 1918-1919 in some countries: the concealment of deaths was common, because they were not heroic like those of the war. Other works reveal the weight that local circumstances played in this process. One such case, discussed by E. Thomas Ewing in chapter 14, was the Soviet Union, where the concurrence of other diseases, famine, civil war, and actions to improve health care and public health obscured the individual memory of the flu pandemic. A particularly interesting chapter is that of Utz Thimm on the memory of the pandemic in the Netherlands, in that it shows how the official public memory of the health crisis was constructed quite differently in Belgium and the Netherlands: it was more present in the latter country than Belgium, where more attention was paid to the First World War. However, the Belgians kept the 1918 flu experience in their individual memory, as the 2009 flu pandemic showed. In addition, in the colonial territories of both countries the experience of the pandemic was worse than in the metropolises, and, given the lack of interest shown in this state of affairs, its memory was preserved through the popular folklore of the colonies. A notable contribution of this book is to highlight the dynamism over a century of the process of the social construction of memory, of oblivion, and of re-awakening to the flu pandemic of 1918-1919.

We believe that the book under review is of great interest for historians specializing in the pandemic that it analyses, but also for researchers on the history of other diseases. Undoubtedly, this work opens up new avenues of analysis of epidemic events, but we also believe that it would be interesting in the future to broaden the fruitful approach it adopts with other case studies, also focused on the 1918-1919 influenza pandemic, in geographical areas less represented in this book, from Europe as well as Latin America, Africa and Asia.
